# Structured Reporting of Superficial Soft Tissue Masses on Ultrasonography: A Closed-Loop Clinical Audit From a Tertiary Care Hospital in Pakistan

**DOI:** 10.7759/cureus.61884

**Published:** 2024-06-07

**Authors:** Muhammad Ahsan Asif, Abdul Subhan Zahid, Ayesha Naseer, Muhammad Ubaid Ullah Khan, Zaeem Bhatti, Muhammad Waris W Khan, Muhammad Faraz K Nizami, Khunsha Shehzad, Usama Afraz Younas, Haseeb Ahmad, Haseeb Mehmood Qadri, Saira Bilal

**Affiliations:** 1 Radiology and Medical Imaging, Jinnah Hospital, Lahore, PAK; 2 Surgery, Lahore General Hospital, Lahore, PAK; 3 Surgery, Jinnah Hospital, Lahore, PAK; 4 Neurosurgery, Punjab Institute of Neurosciences, Lahore, PAK

**Keywords:** research, residents, superficial mass, ultrasound, ultrasound reporting, radiology, pakistan, audit, quality improvement study

## Abstract

Background

Among all the modalities of diagnostic radiology, ultrasonography is considered the least invasive one. However, this benefit usually comes at the cost of its subjective evaluation since it is purely a dynamic diagnostic modality. Thus, instead of ultrasonography, most clinicians usually rely on the report written by the radiologist.

Objective

The objective of this study is to evaluate the clinical practices of ultrasound reporting of superficial soft tissue masses.

Materials and methods

A closed-loop retrospective and prospective study was conducted at the Department of Radiology and Medical Imaging, Jinnah Hospital, Lahore between December 2023 and March 2024. In the pre-intervention phase, a randomly collected sample of 100 ultrasound reports documenting superficial soft tissue masses were included in the study and judged against standard criteria set by the Royal College of Radiologists (RCR). The intervention phase included regular presentations, identification of problems, and designing of preformed reporting forms. Post-intervention assessments were based on the judgment of 100 ultrasound reports in each cycle twice. Intervention and post-intervention assessments were done twice to correct the ongoing practices.

Results

During the pre-intervention phase, the ultrasound reports issued by the department of study showed only 41.5% compliance with the RCR structured reporting guidelines. However, after the first and second post-intervention phases, this percentage increased up to 98.3%. Overall, we observed a compliance difference of 56.5% between the pre-intervention and second post-intervention phases.

Conclusion

Integration of methods, such as briefing the residents on RCR guidelines, displaying parameters, and making structured report templates available, can greatly increase adherence to RCR guidelines for structured ultrasonography reporting. It also greatly enhances the comprehensiveness and reliability of ultrasonography reports for clinicians. Clinical audits should be routinely practiced in the settings of radiology.

## Introduction

Radiology is a constituent part of the diagnosis and treatment of various diseases. Advancements in diagnostic radiology, from the simple X-rays and ultrasound (US) to the more advanced magnetic resonance imaging (MRI) and computerized tomography (CT) scans of internal viscera, have played a huge role in making clinical diagnoses and treatment plans [1]. One of the vastly used non-ionizing imaging procedures in diagnostic radiology is ultrasound [2]. Therefore, answering the precise clinical questions for personalized image-based medicine involves the in-depth knowledge and clinical acumen of radiologists [1].

Radiological reports serve as a crucial source of communication between the referring clinicians and radiologists [1,3]. Since a radiologist has a dynamic ultrasound picture in front of him/her which serves as a direct source of information for the clinical question asked by the clinician, providing a comprehensive report with complete details about the imaging findings and evidence-based differential diagnosis makes an essential part of a radiologists’ job responsibility. It should be free of errors and provide suggestions on patient management. Although the authenticity of all radiological reports is important, it is paramount in ultrasound, the reason being the images available are the only source of representation. The ultrasound report based on images is still subservient to dynamic scans acquired at the time of examination [3]. Radiology reporting serves as the only bridge between a clinician and a radiologist because most clinicians rely on the report rather than the images; therefore, extreme care and a structured reporting pattern should be applied in writing a report [3,4]. Providing reports that are clinically authentic and comprehensive is a learned skill attained through experience, guidance from expert colleagues, and remaining up to date with the present reporting standards [5]. Using structured reporting templates increases the percentage of reports appraised as optimal from 38-70% and the percentage of apposite information found in reports from 38 to 98% [1]. A study concluded that most of the general practitioners and clinicians face difficulty in apprehending radiological terms, therefore, referring and examining physicians prefer structured reports (SR) over free text reports, since it allows them to grasp all the parameters of a radiology report in a better way for making a good clinical decision [5,6].

The training of sonographers to provide clinicians with quality reports that are clinically relevant, accurate, and structured according to a standardized template is crucial. Sometimes it seems easy to bring a change in the clinical settings but the whole process may be too complicated, and what is more mind-boggling in complex settings is to know whether the change will have positive effects or not [7].

To the best of our knowledge and literature search via PubMed and Google Scholar, this is the first prospective audit from Pakistan for the structured reporting of superficial swelling ultrasonography, using the Royal College of Radiologists (RCR) guidelines and with subsequent education and assessment of the participants [8].

## Materials and methods

This closed-loop retrospective-prospective audit was conducted at the Department of Radiology and Medical Imaging, Jinnah Hospital Lahore, a tertiary care hospital, over a period of four months from December 1, 2023 to March 31, 2024 after obtaining approval from the department as an internal audit. 

Department of Radiology and Medical Imaging, Jinnah Hospital, Lahore with 13 radiology residents is a stopover for a large number of patients referred from almost every specialty that is known, providing a range of diagnostic imaging services such as X-rays, CT scans, MRI scans, ultrasounds, contrast studies, and mammograms. There are a total of 13 working stations of Radiology Department spread throughout the hospital, out of which four of them offer the facility of ultrasonography (Table 1). The rest of the stations offer other types of investigations. Each station is run by at least one consultant radiologist.

**Table 1:** Working stations at the Department of Radiology and Medical Imaging, Jinnah Hospital, Lahore.

Description	Numbers
Number of outpatient department (OPD) stations	6
Number of ultrasound working stations	4
Number of emergency stations	3
Total number of working stations in the radiology department	13

Inclusion criteria

Inclusion criteria were as follows: (i) Completely available records of ultrasound of patients, irrespective of their age and gender, and (ii) ultrasounds of swellings of the appendicular skeleton and neck done in the outpatient department on an elective basis.

Exclusion criteria

The exclusion criteria were as follows: (i) ultrasound of patients done in the emergency and (ii) incomplete records of patients.

Pre-intervention observation

In the first month, data collection of random 100 ultrasound reports from the preceding month was done and assessed according to the guidelines of the RCR [8]. A pro forma was generated on Google Forms (Google Inc., USA) consisting of 10 questions as shown in Table 2 and results were generated after the assessment of data.

**Table 2:** Data Items Collected According to the Guidelines of the Royal College of Radiologists.

Criteria	Description	Response	Response
1	Is the anatomical location in the body represented?	Yes	No
2	Is the location in tissue (e.g. epidermal/ dermal/ subcutaneous/ intramuscular) reported?	Yes	No
3	Is the composition (e.g. solid or cystic) of the lesion reported?	Yes	No
4	Is the size in three-dimension reported?	Yes	No
5	Is involved important anatomical structure being reported?	Yes	No
6	Is echogenicity of the lesion being reported?	Yes	No
7	Is the margin of the lesion being reported?	Yes	No
8	Is the sound attenuation of the lesion being reported?	Yes	No
9	Is the vascularity of the lesion being reported?	Yes	No
10	Does the report contain a defined recommendation for further care for equivocal or suspicious cases?	Yes	No

Intervention

At the end of the first month, the results of data after the assessment were displayed to the residents. We conducted two presentations and encouraged discussion among the residents, auditors, and supervisors. Areas of strength and weakness were communicated and reasons were sought. As residents were using blank papers and generic proformas to write the reports manually, we designed a structured ultrasound reporting form making it compulsory for the residents to fill against all data items when making a report as proposed by the RCR.

Post-intervention assessment

During the next month, randomly selected collection of 100 ultrasound reports after intervention was gathered using the newly designed structured reporting form according to the guidelines of the RCR and reassessed. The percentages for the fulfillment of each criterion were calculated individually. There were two rounds of intervention and post-intervention assessment to achieve satisfactory results. Frequencies were calculated and compared to determine compliance and success rates of pre- and post- intervention using the google forms.

## Results

A total of 100 samples were collected in the form of Google Forms. Firstly, the pre-intervention phase was conducted and then standard criteria were provided to trainees. The comparison between these two phases was made and it showed a difference of 2% in anatomical location, 15% in composition, 6% in anatomical structure, and 27% in echogenicity of the lesion being reported. Further improvements were also observed such as 33% in reporting of margins of lesion, 28% in vascularity, 1% in attenuation, and 58% in recommendation. On the whole, about 56.2% improvement was seen in round 1 of the post-intervention phase.

**Figure 1:**
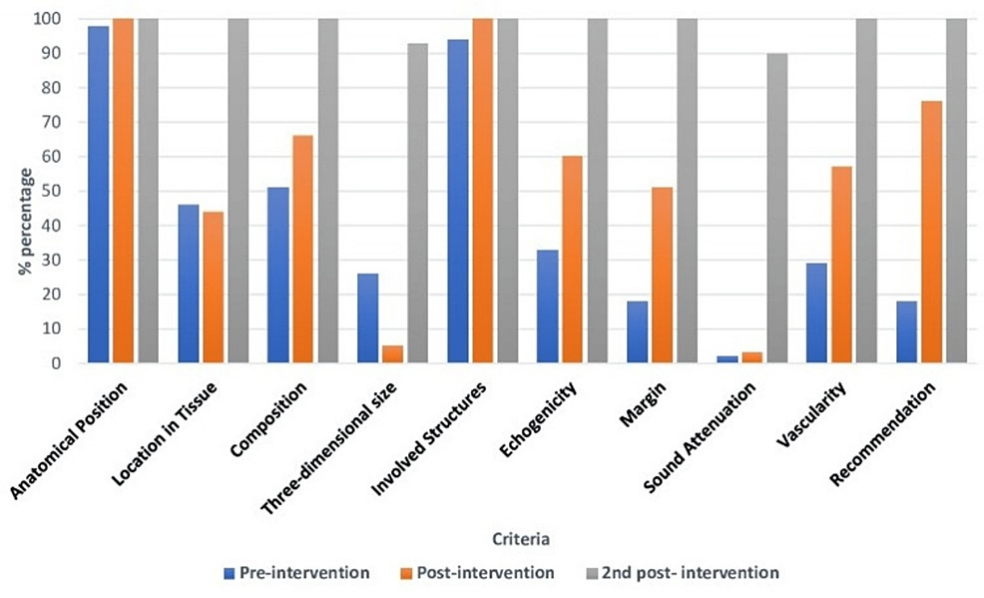
Bar Chart Comparison of Outcomes of Three Phases of Assessment. The Results of the Second Post-intervention Phase Clearly Show Marked Improvement.

The post-intervention phase round 2 showed immense effectiveness in reporting where eight out of ten data items were found to show compliance in 100% of the reports. The rest of the two items including three-dimension size and sound attenuation showed betterment of 67% and 88% respectively. Overall improvement of about 98.3% was observed between the pre-intervention and post-intervention round 2 phase (Table 3 and Figure 1).

**Table 3:** Results of Pre-intervention and Post-intervention Phases.

Sr.#	Data Items	Pre-intervention Phase, n (%)	Post-intervention Phase Round 1, n (%)	Post-intervention Phase Round 2, n (%)	Percentage Improvement between Pre-intervention and Post-intervention Phase Round 2
1.	Is the anatomical location in the body reported?	98 (98%)	100 (100%)	100 (100%)	2 (2%)
2.	Is the location in tissue (e.g. epidermal/ dermal/ subcutaneous/ intramuscular) reported?	46 (46%)	44 (44%)	100 (100%)	54 (54%)
3.	Is the composition (e.g. solid or cystic) of the lesion reported?	51 (51%)	66 (66%)	100 (100%)	49 (49%)
4.	Is the size in three dimensions reported?	26 (26%)	5 (5%)	93 (93%)	67 (67%)
5.	Is involved important anatomical structure being reported?	94 (94%)	100 (100%)	100 (100%)	6 (6%)
6.	Is echogenicity of the lesion being reported?	33 (33%)	60 (60%)	100 (100%)	67 (67%)
7.	Is the margin of the lesion being reported?	18 (18%)	51 (51%)	100 (100%)	82 (82%)
8.	Is the sound attenuation of the lesion being reported?	2 (2%)	3 (3%)	90 (90%)	88 (88%)
9.	Is the vascularity of the lesion being reported?	29 (29%)	57 (57%)	100 (100%)	71 (71%)
10.	Does the report contain a defined recommendation for further care for equivocal or suspicious cases?	18 (18%)	76 (76%)	100 (100%)	82 (82%)

## Discussion

It is a common practice for the authors of this study to conduct quality improvement projects (QIPs) in healthcare centers [9-11]. QIPs should be part of every healthcare center. Upon reviewing the outcomes of our clinical audit, a notable observation emerged regarding the reporting practices concerning soft tissue masses in our radiology department. We found that prior to the implementation of a standardized reporting template, only two parameters, namely, anatomical location and involved anatomical structures, were consistently reported. However, other vital parameters outlined in the RCR guidelines were frequently overlooked. This shortfall in reporting had notable implications for the comprehensiveness and accuracy of information available to clinicians.

The results of the pre-intervention assessment highlighted a lack of familiarity among resident trainees with the structured reporting guidelines outlined by the RCR. The reports produced in this time period were assessed on the basis of these guidelines and were found to lack completeness, consistent with the findings of Botha et al. indicating that deviating from a structured reporting pattern results in subpar reports [12]. This deficiency can probably be attributed to inadequate training in report preparation within the radiology department, aligning with discussions by Wallis et al. [13]. As variability in free-style reporting of ultrasound findings among individuals is influenced by factors such as expertise level, familiarity with reporting standards, and guidance from seniors and colleagues Necas [5]. Hence, the need for structured reporting for superficial soft tissue masses is vital to cover any deficiencies.

In order to make the reports standardized and structured ensuring seamless comprehension by clinicians, we conducted training sessions for resident trainees in the radiology department on the RCR guidelines for reporting. A set of 10 parameters (mentioned in the table) was introduced, with each report expected to adhere to these parameters. To remind and reinforce awareness of these parameters, we prominently displayed the list in procedure stations and rooms. This intervention led to some improvement in the post-intervention phase 1 results which concedes with the results of Edwards et al. indicating that adopting a structured reporting style improves report completeness [3]. Although this intervention led to an overall improvement in the frequency of parameter reporting, it was not a substantial enhancement. One notable finding that caught our attention was the significant difference noted in the frequency of reporting of the three-dimensional size of the mass, which dropped down by 80% in the 1st post-intervention assessment. Further investigation revealed that recalling all parameters while preparing reports amidst increased patient loads was challenging, highlighting inefficiencies in the reminder charts displayed to radiologists and the time-consuming nature of the task to prepare such SR. This finding aligns with those of dos Santos et al. [1].

In the subsequent phase of implementing the structured reporting guidelines, we introduced report templates that listed the parameters. The assessment following this, demonstrated that the structured templates substantially improved the reporting frequency of each parameter, with significant improvement noted in the sound attenuation of the lesion and inclusion of recommendations for further care. This finding is consistent with those noted by Fraser et al., indicating that structured templates notably enhance ultrasound reporting [14].

Superficial soft tissue masses present a diverse range of potential diagnoses, often identified through thorough history-taking, clinical examination, and imaging assessment [15]. Ultrasonography is the preferred imaging method for evaluating these masses, often prompted by specific clinical queries from referring clinicians. Subsequently, radiology reports are provided to address these queries and outline details of the masses in question. Given that clinicians rely on these reports for treatment planning, it is crucial for reports to encompass relevant details, address clinical inquiries, and offer recommendations for further management and imaging. As structured reporting encompasses all the crucial aspects of such a mass that can be assessed using ultrasonography, as mentioned by Jacobson et al. and RCR guidelines, it can greatly improve the understanding of the lesion and its management [15].

While efforts were made to train the resident trainees on structured reporting guidelines, the effectiveness of this training may vary among individuals. Some trainees may have grasped the guidelines better than others, eventually influencing the consistency of reporting.

Limitations

There were several limitations to this study. The Hawthorne effect is the first in the list. The healthcare providers were aware of the fact that they were being observed. This can lead to a temporary adherence to the protocols and parameters being introduced, resulting in an increase in structured reporting. The audit's findings are based on observations within the radiology department of Jinnah Hospital, Lahore. It's possible that the results may not be generalizable to other departments or institutions due to variations in workflow, patient loads, or resources.

Clinical recommendations

The radiology department is encouraged to continue using structured reporting for ultrasound reports and training residents on the guidelines set by the RCR to ensure consistent, standardized reporting. Keeping report templates that list key parameters can enhance each parameter reporting and capturing vital information. Modifying reminder charts can help radiologists remember crucial parameters while preparing reports during times with high patient influx. Follow-up audits should be conducted to maintain the improvements seen during the audit period and to get clinicians' feedback. The findings from the audit conducted at Jinnah Hospital, Lahore could be useful if applied to radiology departments in other institutions to see their generalizability. Further research is necessary to improve interventions and evaluate their long-term effects addressing issues like a drop in the 3D size measurement parameter post-intervention, the Hawthorne effect, and differences in training effectiveness. Radiologists should aim for reports that also assist in patient management in ultrasound testing where images play a crucial role. The department should consistently identify strengths and weaknesses, understand their causes and implement measures, for quality enhancement.

## Conclusions

Structured reporting is crucial to enhance the comprehensiveness and accuracy of ultrasound reports concerning superficial soft tissue masses. While the initial interventions, such as briefing the resident trainees on RCR guidelines and displaying parameter lists, showed modest improvements, the introduction of structured report templates notably improved the reporting frequency and adherence to the parameters. This finding highlights the importance of structured reporting of superficial soft tissue masses in optimizing the diagnosis, planning out management, and further investigations. However, limitations such as the Hawthorne effect, training effectiveness, and lack of generalization should be acknowledged, warranting further research into refining the interventions and assessing their long-term impacts. Ultimately, the integration of structured reporting guidelines for superficial soft tissue masses into routine practice can improve diagnostic clarity and facilitate informed clinical decision-making in the evaluation and management of these lesions.
